# Instrumentation for Vibrational Circular Dichroism Spectroscopy: Method Comparison and Newer Developments

**DOI:** 10.3390/molecules23092404

**Published:** 2018-09-19

**Authors:** Timothy A. Keiderling

**Affiliations:** Department of Chemistry, University of Illinois at Chicago, Chicago, IL 60607-7061, USA; tak@uic.edu

**Keywords:** vibrational circular dichroism, instrument design, IR polarization optics, absolute configuration, biopolymer secondary structure

## Abstract

Vibrational circular dichroism (VCD) is a widely used standard method for determination of absolute stereochemistry, and somewhat less so for biomolecule characterization and following dynamic processes. Over the last few decades, different VCD instrument designs have developed for various purposes, and reliable commercial instrumentation is now available. This review will briefly survey historical and currently used instrument designs and describe some aspects of more recently reported developments. An important factor in applying VCD to conformational studies is theoretical modeling of spectra for various structures, techniques for which are briefly surveyed.

## 1. Introduction

Vibrational circular dichroism (VCD) measurements were developed in the 1970s in several labs for studies of conformation and configuration of chiral molecules. Extension of CD measurement capability to the infrared (IR) region was long sought and anticipated, due to the large number of resolved, localized chromophores accessible in the IR, which offered the potential of added structural information. However, early VCD instrumentation development was hindered by the lack of appropriate IR polarization and detection technology. Polarized spectra of chiral molecules were routinely studied in the visible or ultraviolet (UV), using optical rotatory dispersion (ORD, sensing the rotation of linear polarization, but primarily non-resonant, measured away from the absorbance band) and circular dichroism (CD), which is the differential absorbance (ΔA = A_L_ − A_R_) complement to ORD (measuring spectra of resonant transitions) [1,2]. CD reflects contributions of the specific states being excited, but visible or UV spectral bands arise from transitions between electronic states whose spectral bands are generally quite broad, especially for organic molecules, since they are often vibronic in nature, and often represent overlapped, delocalized transitions. The more resolved and localized vibrational transitions in the molecular ground state that correspond to conventional IR spectra thus provided an attractive target for the development of new, IR-focused CD instrumentation.

Experimental realization of vibrational CD (VCD) has faced significant difficulties, since VCD of fundamental transitions has been expected to be (and generally is) very weak in intensity (ΔA/A~10^−4^–10^−5^) [3,4,5,6,7,8]. Beyond its having low differential absorbance (ΔA), there are fundamental problems in the theoretical prediction of VCD, which have been now largely solved, but analyses of underlying theoretical developments is beyond the scope of this instrumentation-focused review [6,9,10]. VCD was thus challenging for both measurement and analysis, and its experimental development depended on advances in IR technology that overcame some of the barriers.

Initial VCD instruments were constructed in the same manner as conventional UV-vis CD spectrometers, but with various components altered to provide functionality in the IR [11,12,13,14,15]. Typical CD instruments have bright broadband sources and dispersive optics that utilized a sequentially scanned prism or grating to select the wavelength bandpass that passed through a slit [16,17]. To operate in the IR and maximize signal-to-noise ratio (S/N), many alterations to conventional CD designs were needed [18]. IR spectrometers use black-body light sources whose total output increases with temperature, but this option for increased light intensity has a smaller impact in the more important mid-IR range. Reflection-based optics and grating monochromators are generally advantageous to avoid chromatic effects, spectral transmission, and dispersion limitations of lenses and prisms in the IR. Although materials are limiting for any device that the beam must pass though, e.g., sample cells, they are critical factors in the components used for creating the polarized light. Most VCD spectrometers use grid polarizers, holographically formed on IR transmitting, isotropic substrates. These have a wide angular aperture, leading to more convenient optical designs than would be possible with crystal prism polarizers, although the latter do have higher extinction ratios. Semiconductor detectors are used both for sensitivity, needed to detect very low amplitude modulations, and for speed of response, required by high modulation frequencies.

Initial VCD instrument designs incorporated such changes [11,12,13,15] and were further refined in several laboratories [18,19,20,21,22,23,24,25,26]. The pioneering work of Nafie and co-workers in the development of Fourier-transform-based VCD instrumentation shifted the methodology, and such instruments now dominate the VCD field [19,27,28,29,30,31,32,33,34,35]. While those original designs were subsequently modified in various labs [36,37,38,39,40], the work of his Syracuse group, and the evolution of their concepts into commercial instrumentation, have revolutionized VCD measurements and made them truly widespread [5,6,29,41,42]. As a result, measurement of VCD for a wide variety of molecules, typically for samples in solution phase, is now routinely possible over the near IR and much of the mid-IR, as has been thoroughly reviewed in a number of formats over the past two or more decades [5,6,8,20,43,44,45,46,47,48,49,50]. Consequently, this review will focus on VCD instrumentation and their developments that have enabled numerous applications to be realized.

## 2. Common Features of VCD Instruments

Dispersive and Fourier transform (FT) VCD, just as ordinary IR and FTIR, differ in the way intensity variations at the optical frequencies, the two basic spectral variables, are determined. Dispersive VCD instruments use a grating scanned through the required wavelengths to sequentially record the spectrum. FT-VCD instruments use a Michelson interferometer whose moving mirror varies the interference of two beams recombined at a beam-splitter; as a result, the detector senses all wavenumbers simultaneously (multiplex advantage). However, these are weighted by the mirror positions, which can then be used to convert to wavenumber representation by Fourier transformation. Dispersive instruments use time-response averaging and filtering to improve S/N, while rapid-scan FT-VCD averages many successively measured interferograms for S/N enhancement. The noise level in dispersive VCD experiments varies inversely (and somewhat non-linearly) with transmission at each wavenumber, while in FT-VCD the noise is more dispersed (but due to intensity normalization, see below, there is still an impact of the transmission spectrum on S/N). Both types of instrument work well for their designed purposes. FT-VCD can produce high quality spectra over the entire mid-IR, typically from ~2000 to ~800 cm^−1^, with good resolution (often ~4 cm^−1^ is used) in a reasonable time, due to its multiplex advantage, as has been extensively reviewed [5,6,8,18,43,44,45,46,47,49,50]. (Near-IR and mid-IR FT-VCD spectra can be measured under the same conditions, but there are advantages to obtaining them separately.) Such broad-spectral measurements of VCD work well for chiral organic molecules in non-polar solvents (fewer absorbance interferences). As a consequence, FT-VCD dominates the commercial market, where most applications have been targeted at absolute configuration determinations and conformational analyses, typically of moderately sized molecules, which can often be studied in organic solvents. Dispersive VCD instruments are too slow to be efficiently used over a broad wavenumber region but have been optimized for high sensitivity in narrow frequency regions to give excellent S/N with relatively flat baselines at somewhat lower resolution [43,49,51,52]. For many biopolymer studies, particularly for peptides and proteins, there are only a few accessible broad bands, and ideal enantiomer baseline samples are not possible. In such cases, these dispersive VCD constraints fit the goal, and the corresponding instruments provide excellent spectra.

Dispersive and FT-VCD instruments use conventional black-body light sources, e.g., glowbars or the like, but these have been modified for targeted purposes. The monochromator or interferometer output is passed through a wire grid polarizer (in all current designs), and a photo-elastic modulator (PEM, now typically containing a ZnSe optical element), to yield the modulated (elliptically) polarized light. Since the degree of circular polarization from a PEM (which only has λ/4 wave retardation at one wavelength) varies over the spectrum, the amplitude must be corrected by calibration. After passing through the sample, the light beam is focused on a detector, typically a liquid-N_2_-cooled diode photoconductor, such as Hg_1−x_(Cd)_x_Te (MCT), or alternatively photovoltaic InSb for the near IR.

The detector signal in these types of VCD instruments is doubly modulated, at the PEM frequency (ω_M_, typically ~30–50 kHz) and secondly at lower frequencies (<kHz) due either to a chopper (ω_C_, dispersive) or variations in the interference from the mirror motion (FT). Modulations generating an AC signal for transmission are used since photodiodes are not very sensitive for DC detection. The signals at these two modulations can be electronically separated by filters into two channels, one for the transmitted (I_trans_) intensity and one for polarization modulated (I_mod_) component due to the differential absorbance, ΔA. In a dispersive instrument, DC voltages correlated to the amplitudes of both modulations are obtained using lock-in amplifier demodulation methods and are digitized and processed in a computer. In FT-VCD, the computer digitizes the time-varying interferogram and Fourier transforms it to yield I_trans_. Another interferogram, resulting from processing the high frequency modulation (ω_M_) with a very low time constant lock-in or with digital signal processing, is then transformed to yield I_mod_. In both types of instrument, the raw VCD signal is proportional to I_mod_/I_trans_, which normalizes out the signal variation due to changes in instrument transmission. Once corrected for gain and sensitivity, this ratio provides a signal proportional to ΔA = A_L_ − A_R_. (It can be noted that errors or non-linearities in measuring I_mod_ or I_trans_ can result in apparent signals that reflect the transmission spectrum, or VCD artifacts, a point returned to later.) For small ΔA values (the normal VCD situation),

I_mod_/I_trans_ = (1.15 ΔA) J_1_(δ_0_) g_a_ (1)

where J_1_(δ_0_) is the first order Bessel function at δ_0_(λ), the modulator’s maximum phase retardation at the wavelength λ, and g_a_ is an amplifier gain factor. Using a birefringent plate and polarizer pair to form a pseudo CD signal: I^c^_mod_/I^c^_trans_, one can create a calibration spectrum and use it to eliminate gain factors and the J_1_(δ_0_) dependence [12,18]. In principle, this signal provides an absolute calibration:

I^c^_mod_/I^c^_trans_ = [J_1_(δ_0_) g_c_ sin α_B_]/[1 + J_0_(δ_0_) cos α_B_] (2)

where α_B_ is the retardation of the plate, J_0_(δ_0_) is the zero-order Bessel function at the maximum retardation of the modulator, and g_c_ is the altered gain used for the calibration scan. Since the retardation of a multiwave plate is strongly wavelength-dependent, at those λ^c^ values for which

α_B_(λ^c^) = (2n + 1) π/2, (3)

Equation (2) simplifies to

I^c^_mod_(λ^c^)/I^c^_trans_(λ^c^) = ±[J_1_(δ_0_) g_c_] (4)

so that

ΔA = {[I_mod_/I_trans_]/[I^c^_mod_/I^c^_trans_] }/{1.15 (g_a_/g_c_)}. (5)

It should be noted that, since birefringent plates are angle sensitive, one should measure the calibration with the same optical alignment and aperture as used for the samples. A simpler calibration can be obtained by comparing the measured sample VCD signal with that of a standard sample [18,38,40,43,49,51,53,54]. After averaging spectral data sets and subtraction of baseline spectra obtained under the same conditions and with the same sample cell (or its equivalent, since stray birefringence in the cells can cause baseline variations), the processing is complete. Smoothing of spectra or conversion to molar quantities, e.g., Δε(λ) = ΔA(λ)/bc, where b is the path length in cm and c is the concentration in moles/L, can also be done.

## 3. Dispersive VCD Specifics

The earliest VCD spectrometers were built for near-IR (C–H stretch and related modes) and used W-I quartz-halogen lamps as bright, high temperature sources, all reflecting optics, self-made PEMs, and InSb photovoltaic detectors for sensitive detection without the need for a bias voltage [11,12,13,14]. The next designs were conceptually similar but varied from this in some details, using alternate commercial modulators (CaF_2_ or other from Hinds, Inc., Hillsboro, OR, USA), sources (EIMAC Xe arc with sapphire window) and incorporating CaF_2_ lenses after the sample, for straight-through optical design [14,20,21,55,56,57,58,59]. These instruments were extended to the mid-IR by use of alternate sources, MCT detectors, and ZnSe optics, with a practical low wavenumber limit appearing to be ~800 cm^−1^ (where normal MCT detectors cut off). While other detectors can go lower, ZnSe cuts off at ~600 cm^−1^ and going below that is very challenging due to materials limitations [18,21,26,60]. Variations in detectors, signal processing, and optical configuration provided minor improvements, and have even enabled measurements of VCD for overtone and combination bands in the near IR, but most instrumentation developments in the last two decades focused on FT-VCD instrumentation [18,20,23,24,25,26,27,61,62,63,64].

**Figure 1:**
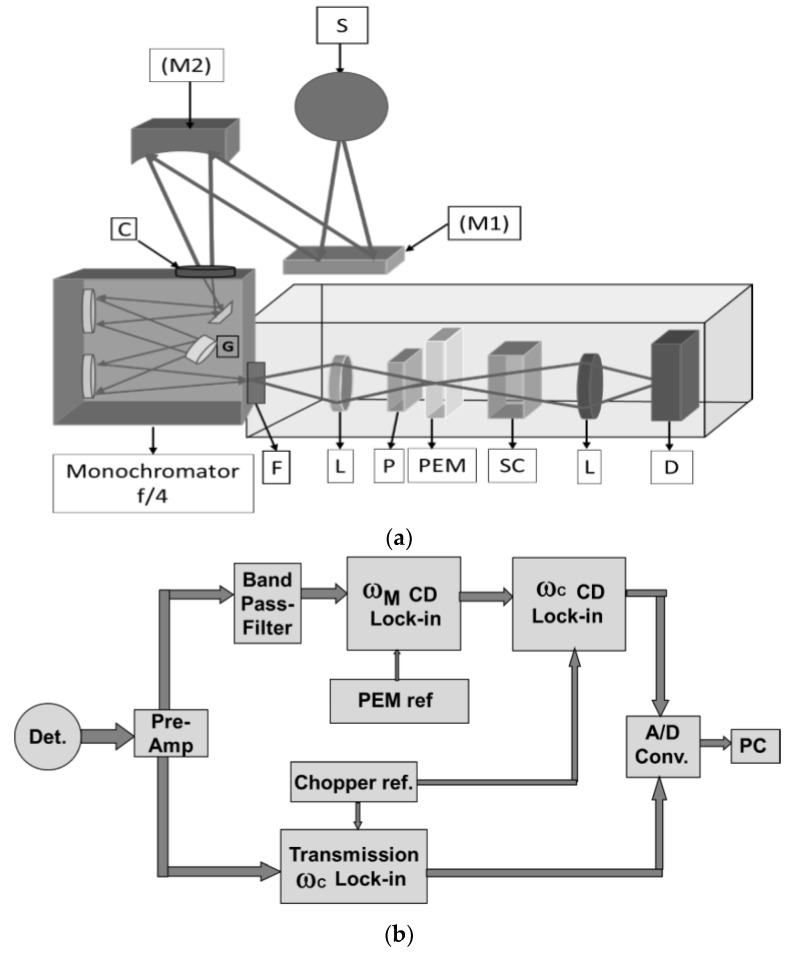
(**a**) Schematic diagram of the optical components of an improved dispersive vibrational circular dichroism (VCD) spectrometer, optimized for peptide and protein studies. S-Carbon-rod light source in cooled housing; M1: Flat mirror; M2: focusing mirror; C: rotating wheel chopper (150 Hz); G: exchangeable monochromator grating (on turret, 150 or 200 mm/groove); F: optical long wave pass filter; P: wire gird polarizer (BaF_2_); PEM: photoelastic modulator (ZnSe); SC: sample cell; L: lens; D: MCT detector. (**b**) Schematic diagram of the electronics components of the new dispersive VCD spectrometer. Each component is designated in the diagram.

The most recently constructed dispersive instrument for obtaining fundamental band VCD spectra was built at UIC and is optimized for biopolymer studies [51,52]. A water-cooled, resistively heated (2500 K) carbon rod, matched to the slit size, provides high intensity IR radiation (substantially brighter than a glowbar) [18,21]. The light is collected and passed through a chopper (ω_C_~150 Hz) that modulates the transmission signal and is then focused on the slit of a 0.3 m focal length, ~f/4 monochromator (SpectraPro2300i, Princeton Instruments, Acton, MA, USA). With a 150 g/mm grating blazed at 6 μm, we vary the slits to provide an 8–12 cm^−1^ resolution, depending on the sample, but the monochromator can hold and switch between three gratings for use in different spectral regions. An optical schematic is shown in Figure 1a. As compared to previous designs, the most important S/N improvements of this instrument were its higher optical throughput (faster, f/4 monochromator) and increased MCT sensitivity (D*), due to its having a narrow band pass (cut off ~8 μm).

This revised instrument was optimized for study of peptides and proteins in both the amide I and II regions and has no reflective optics after the fast monochromator exit slit. Its output is instead collected with a BaF_2_ lens, polarized and modulated by passing through a BaF_2_-substrate holographic wire grid polarizer and a 37 kHz ZnSe PEM (Hinds Instruments) and focused on the sample. With another BaF_2_ f/1 lens, the light from the sample is refocused onto the high D* MCT detector (Infrared Associates). Use of a small “array” detector can improve S/N. These form a stack of three or four 2 × 2 mm MCT elements (plus parallel preamps that feed a summing amplifier), whose geometry reflects the shape (aspect ratio) of the slit and its focused image through the sample and on the detector [43,49].

For signal processing, the transmitted intensity, i.e., the detector signal in-phase with the chopping frequency, ω_C_, is converted to a DC voltage, V_trans_, by use of a lock-in amplifier whose output is digitized and passed on to the computer. A second lock-in (Stanford, SR830) detects the polarization modulation intensity, I_mod_, which is phase-referenced to the PEM frequency, ω_M_, but uses a very short output time constant (<ms). This ω_M_ lock-in output can then be demodulated again using a third lock-in referenced to the chopper, ω_C,_ to provide V_mod_. This double demodulation concept is based on the dependence of the raw CD signal on the light-level, so it is doubly modulated, but noise and other electronic interfering signals that appear in the ω_M_ bandpass do not depend on the light. V_mod_ is digitized and ratioed in the computer to V_trans_ using a LabVIEW software routine written in-house [51]. The overall design is schematically shown in Figure 1b. Digital normalization [23,24,25] has more dynamic range (for more highly absorbing samples) than earlier dynamic normalization schemes that used a feedback loop providing variable gain [11,12,15,18,20,21,57]. As noted above, more reliable measurement of I_trans_ with digital processing can lead to a reduction of false signals (artifacts) correlated to the transmission. Simultaneous collection of the IR absorbance and VCD in each scan are natural characteristics of this digital approach that enhance control of measurement and sample stability.

## 4. FT-VCD Design

FTIR-VCD spectrometers have been developed in several labs [18,27,28,29,30,36,37,39,47,53,54,65,66,67,68,69,70,71,72,73] but all are based on the initial concepts of Nafie and co-workers [32,33,74] and have been extensively reviewed [5,18,40,43,46,47,75]. They commonly utilize a commercial FTIR (either a separate instrument or an internally integrated interferometer) to which polarization optics as well as processing electronics and software are added for purposes of VCD measurement. High sensitivity commercial VCD spectrometers or attachments to FTIRs are now available from several vendors, (e.g., BioTools, Bruker, and Jasco), so there has been little impetus for research labs to continue developing FT-VCD in recent years, although in-lab constructed FT-VCD instruments are still operable.

**Figure 2:**
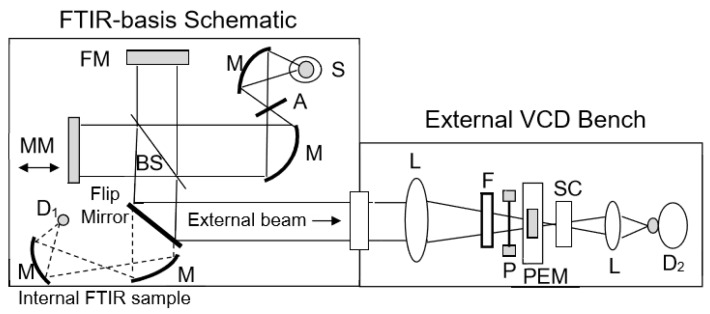
Schematic of the main optical components of a typical FT-VCD instrument constructed by coupling a VCD accessory bench to a commercial FTIR. These include (S) a light source, (M) collimating mirrors with (A) aperture restriction, (FM) a fixed mirror, (MM) a moving mirror, (BS) and a beam splitter, plus normal sampling using separate focusing mirrors and (D_1_) an internal detector for independent transmission FTIR. A schematic of an external VCD bench includes (L) a sample focusing lens, (F) a filter, (P) a wire gird polarizer, (PEM) a photoelastic modulator, (SC) a sample cell, (L) a detector focusing lens, and (D_2_) a MCT detector for VCD.

The interferometer used is typically of rapid-scan design, incorporating a KBr beam splitter coated for mid-IR use and a glowbar or other heated ceramic source. Its output beam, often employing a collimated or weakly focused beam to reduce artifacts, [39,53,72] is directed through a polarizer (wire grid) and PEM (ZnSe) and on to a sample, after which it is focused onto an MCT detector, typically with a ZnSe lens [5,18,27,35,39,46,47,72]. This concept is schematically illustrated in Figure 2 for the case of an in-lab constructed instrument based on an FTIR with a separate VCD accessory. Use of lenses (ZnSe or BaF_2_) as opposed to more conventional mirrors for focusing in the sample part of the optical train have advantages for control of artifacts, which can be a problem for FT-VCD [39,72]. The optics are functionally similar for most instruments, but they do vary in terms of sampling and electronics. While the BioTools and Jasco instruments are dedicated VCD spectrometers, with integral sample compartments, the Bruker design utilizes one of its regular FTIRs with a separate VCD attachment that contains the polarization optics, sample holder and detector. This latter can also be modified for linear dichroism and reflection measurements and resembles the separate sampling used in most lab-constructed instruments, such as ours. (Jasco and BioTools also market separate VCD FTIR accessories, the latter designed for coupling to FTIRs from other manufacturers. While other FTIR companies have offered such designs, they have had less impact on the market to date.) Optical filters (e.g., 1900 cm^−1^ cut-off low-pass) are often used to isolate the mid-IR and avoid saturation and non-linear response of MCT detectors, which can result from the broad source spectrum being delivered to the detector without sufficient attenuation. Sample cell design and solvents can also limit band pass and consequently minimize saturation effects. Utilizing its corner-cube interferometer design, the BioTools instrument incorporates a second source, which by out-of-phase interference of the two beams can decrease MCT saturation and increase S/N (see below) [29]. Control of non-linear response of the detector is important for minimizing artifacts. In general, such detector issues can be identified from the appearance of a non-zero transmission (single-beam) baseline in regions where there should be no detectable response (e.g., below the detector cutoff wavenumber). These are common issues for FTIRs with MCT detectors, but in our experience, limiting the spectral range to below 2000 cm^−1^ with an optical filter, combined with light losses due to other optics and the sample in the beam, can adequately address the problem.

The output of the VCD detector is normally amplified, filtered, and split, allowing the low-frequency modulation due to the moving mirror (few kHz or less) to be digitized for conventional FT processing of the transmission, I_trans_. The ω_M_ components are shifted to DC with a lock-in (using an ~ms time constant), but this signal has sidebands, due to the moving mirror, that form another interferogram, which, when Fourier transformed, represents the VCD modulation signal, I_mod_. Since the I_mod_ interferogram is obtained by passing the signal through a time constant filter in the lock-in output channel, it is important to use moderate mirror speeds, which are slower than normal for MCT detection. In terms of 1/f noise, this is not a problem since the I_mod_ detection is initially done at ω_M_. A transferred phase correction, obtained by measuring a signal that has only one sign and is processed in the same way as the sample VCD, through all the same electronics, is used. Alternatively some other method, is needed, since I_mod_ is a differential signal that has +/− signs, and the normal (Mertz) phase correction is designed to yield only positive spectra [18,38,39,65]. The I_mod_/I_trans_ ratios are subsequently used to determine the VCD spectrum [18,27,39,53,54,65].

Near-IR FT-VCD can also be measured and can have high S/N if an InSb detector is used, but, due to the shorter wavelengths, this requires that the instrument be able to digitize higher frequency modulations, or one must use slower mirror scan speeds [28,30,31,54,66,68,76]. VCD can be measured with step-scan FTIR designs, but this approach has shown no advantages to date, except that it provides a setup compatible with different modulation methods, particularly those having lower frequencies [38,54,66,70]. Digital signal processing (DSP) has been realized with a unique step-scan design, without great improvement in VCD measurability (see below) [70,77]. The updated BioTools and Jasco rapid-scan instruments claim improved S/N due to their versions of DSP (see below), and, of course, modern lock-ins use DSP technology to improve filtering and dynamic range.

## 5. Comparison of Methods

FT-VCD spectrometers dominate the market and now offer high sensitivity and a relatively good control of baseline artifacts. For studies of smaller organic molecules in nonpolar solvents, and determination of absolute configurations, the advantages of collecting the entire spectrum simultaneously and of using higher resolution make FT-VCD the optimal design choice. Such studies demand measurement of spectra over a wide wavenumber range that can then be compared to theoretical simulations, with the goal of obtaining absolute stereochemistry determinations as well as carrying out conformational analyses. Since FT-VCD accesses the entire mid-IR (~800–1800 cm^−1^) in a single experiment, often in just a few minutes, and standard “IR solvents” (e.g., CCl_4_, CS_2_, and CHCl_3_) are relatively free of interferences over this region, FT-VCD is an excellent match to the dominant use of VCD for studies of moderate sized chiral organic molecules. Larger, fluxional molecules are more challenging, due to extensive conformational averaging, which tends to reduce VCD amplitudes, and insoluble molecules are even more difficult.

For analyses of structures and conformations by theoretical modeling, it is desirable to express observed VCD in molar units, i.e., as ε and Δε, which requires the independent determination of concentrations. This is relatively straightforward for pure samples and dilute solutions, such as one can often obtain with smaller molecules and “IR solvents”. FT-VCD spectra are naturally smooth, since the FT process samples the data at the actual spectral resolution, but baseline fluctuations can appear as real spectra and confuse interpretation. However, with “IR solvents” or neat samples, the S/N of modern FT-VCD measurements for relatively rigid chiral molecules is so high that this is not a significant issue. For smaller molecules where data collection over the entire spectra region is very efficient with FT-VCD, this method is mature and will continue to dominate the field, and dispersive VCD cannot compete in terms of either time scale or resolution.

While very few dispersive VCD instruments remain active, there is a limited role for them in terms of focused studies of biomolecules in aqueous solution, where the bands are broad and, due to solvent spectral windows, there is only a limited useful spectral range that can be scanned. The main FTIR throughput and multiplex advantages have much less impact in such cases. Since dispersive VCD utilizes all its measurement time/effort on just the region scanned, one can focus the scans on a single band (especially for proteins). The instrument can have very high optical throughput for such broad bands, since low resolution means one can use larger slits that can match the source geometry, and the dispersive design allows use of higher power light sources without concern for detector saturation [22,43,49,51,52,78].

Dispersive VCD spectra are scanned slowly, typically with a time constant, τ~10 s. For ~10 cm^−1^ resolution, it takes 15–30 min to measure amides I and II, which should be repeated and averaged to establish reliability and obtain optimal S/N [23,24,51,52,61]. While FTIR-VCD rapidly samples the full spectrum, many scans (typically thousands) must be averaged to obtain adequate S/N, particularly for aqueous phase biopolymers. A greater spectral range means dispersive VCD would take more time, but this is not the case for rapid-scan FTIRs. By contrast, if higher resolution is needed, FT-VCD does require longer mirror scans; consequently, more time is required for the same number of scans and S/N is reduced, which requires more averaging, so that the required time increases non-linearly. Neither style of VCD measurement is now compatible with monitoring fast (<sec) kinetic events. However, with tunable laser sources (see below), one could envision obtaining relatively rapid (sub sec) single wavenumber VCD response as a function of time. Dispersive VCD and step-scan FT-VCD are compatible with alternative (slow) modulation styles that might allow access to different spectral regions [38]. Since dispersive VCD is a direct measurement, it is conceptually easier to interpret and troubleshoot, particularly for spurious signals (artifacts or noise) [23,51,52].

Baseline correction and artifact rejection have long been an issue for VCD, due to its intrinsically weak intensity. An ideal baseline should be obtained with a racemic (or opposite enantiomer) sample measured under identical conditions. While often possible for synthesized small molecules, this method is not compatible with many natural product or biomolecule studies. We obtained useful dispersive VCD baselines for aqueous biopolymers using just solvents in the same or an identical sample cell, and then subtracted it from the sample VCD. Use of different cells may result in a baseline offset, but usually a consistent shape is obtained. For non-aqueous samples, this may not work as well, especially for high refractive index solvents. Creating a “racemic sample” in the same solvent so that its absorbance is similar to that of the target sample (e.g., employing simple ketones or amides, etc., for chiral ones) may offer an alternate means for simulating a baseline. Baseline correction has been a challenge for some FT-VCD instruments, but Nafie’s dual modulator design can dramatically improve baselines (see below) [71,79].

**Sampling considerations.** Most VCD studies are done on solution phase samples, which for non-aqueous samples employ standard IR cells of fixed or variable path lengths using KBr or other isotropic (minimal birefringence) salt windows to enable broad spectral access. Neat liquids can also be studied in this way but require much shorter path lengths. In general, the absorbance of the bands to be measured should be <1, and optimally ~0.5. For aqueous samples, it is critical that windows not be hydroscopic, so CaF_2_ or BaF_2_ are most commonly used. Cells consisting of two thin CaF_2_ windows separated with a spacer and sealed using O-rings into a brass frame have proven to work well for biopolymer studies and has aided in minimizing baseline artifacts in our instruments [43,49]. Thin cells, short pathlengths, and low indices of refraction (solvent and window) seem to be useful characteristics for reducing artifacts.

This cell design can also be used for temperature variation studies. In our lab, the cell in its brass ring is inserted in a jacketed brass holder through which water from a bath is pumped to vary and control the temperature [49]. By substituting alternate bath fluids (e.g., glycol) for water, lower or higher temperatures can be accessed. Commercial cell holders are also available from various vendors that use thermoelectric, Pelltier temperature controllers and can often access temperatures from 0 to 100 °C, which is sufficient for aqueous and biopolymer samples. VCD studies of peptide or nucleic acid conformational change caused by increases in temperature can parallel similar studies with CD, fluorescence, and IR. The virtue of VCD is sampling a different interaction than seen in FTIR, in a more conformationally discriminatory manner, and offering different structural emphases than in ECD or fluorescence. Such probing of the conformational equilibrium can help detect intermediates in the folding pathway.

The VCD of peptides or proteins in D_2_O can be obtained with concentrations of ~10 mg/mL, or even a low as ~1 mg/mL, using path lengths up to ~100 μm [43,49]. Such samples are relatively dilute, so it is possible to directly measure their ECD in the same cell to demonstrate continuity of biopolymer conformation with even more dilute ECD and fluorescence experiments. If one is concerned about issues of H/D exchange, or creating a mixed level or protonation/deuteration, or if the sample cannot be lyophilized to effect a more complete exchange, VCD of peptides or proteins can, of course, be measured directly in H_2_O-based solutions. However, for a good S/N, very short (~6 μm) path lengths (due to the HOH bending vibration at 1650 cm^−1^, which directly overlaps the peptide amide I band) and much higher concentrations (>50 mg/mL) are often needed with H_2_O [80,81,82,83]. Under such conditions, intermolecular interactions can change the conformation, particularly for peptides with extended structures. Added studies that vary concentration can help establish the reliability of such results. IR-based biomolecular experiments tend to have both inaccurate concentrations and path lengths, so their VCD is often normalized to the absorbance maximum or to the area of a common band (e.g., amide I) for comparison of relative intensities in different samples.

Gas phase samples can also be easily studied with VCD, but due to vapor pressure limitations, this is only practical for small molecules and has been applied in only a few circumstances [84,85,86]. Conventional multipass cells will cause problems for VCD since reflections can introduce a polarization in the beam. The macro-size of VCD beams are also challenging for such long-pathlength samples, but that has been solved in other IR experiments. Polavarapu has addressed rotation-vibration effects, and has also predicted a rotational analog to VCD, which has become a topic of current theoretical interest [86,87,88]. With magnetic VCD (see below), such rotationally resolved spectra are commonly measured.

Solid samples can also be studied, varying from classic IR methods of mull or KBr pellet preparations to films made by an evaporating solvent, but these can lead to various artifacts, and care must be taken in interpreting the results of such VCD measurements [58,89,90,91,92]. Particulate scatter is detrimental to VCD, causing some loss of polarization, but due to the longer wavelengths in the IR, this is much less a problem than for ECD. More recently, measurements of VCD for molecules adhered to nanoparticles have been reported [93,94]. We and others have succeeded in measuring spectra of aggregated samples, fibrils, and films, as well as of proteins in membrane vesicles [52,95,96,97,98,99,100]. In such non-isotropic samples, the spectra must be controlled for distortion due to macroscopic characteristics of the sample, for example by measuring the sample at different angles to test for orientation dependence. Commercial sample rotators are available to minimize such effects. In particular, enhanced VCD of fibrillar samples have been shown to be diagnostically useful and morphologically sensitive, although the origin of the enhancement is still open to investigation [95,96,97,98,100,101,102,103,104,105,106,107].

**Less common measurements.** One aspect of improving spectra with the same instrumentation is to increase the spectral resolution, but if the spectra themselves are broad, the spectrometer resolution itself will have little impact. Changing the solvent to one with reduced intermolecular interactions (e.g., eliminate H-bonding) can have modest effects on bandwidths, but varying the temperature such as described above with conventional controllers has little impact. One approach to improving the sample’s spectral resolution is matrix isolation, by which molecules are trapped in a frozen gas matrix and their spectral transitions typically become sharper both by less mixing with low frequency modes and/or by being trapped in one or a few favored conformations. Stephens and co-workers reported matrix-isolated VCD for α- and β-pinenes in the C–H stretching, near-IR region and showed development of a spectral pattern different from the room temperature solution results, with more resolved features as well as a sharp enhancement in intensity [108]. Subsequently Henderson and Polavarapu [109] showed that the mid-IR VCD of matrix-isolated α-pinene measured on an FT-VCD at a 1 cm^−1^ resolution showed dramatically sharper lines and some new features. Couplets in particular were enhanced due to the increased resolution. This method is having a renaissance, with Vass, Xu, Merten and co-workers using improved instrumentation and reporting studies of propylene oxide, methyl lactate, and small alcohols correlated with spectral simulations to determine structures of isolated molecules or of their complexes in the matrix [110,111,112].

While VCD was developed and is almost solely used for study of chiral molecules, all molecules can develop a VCD spectrum if the samples are placed in a magnetic field that is co-linear with light propagation. This is termed magnetic VCD (MVCD) and is the vibrational analog of magnetic CD, a well-developed technique used for characterization of excited electronic states. A number of experimental and studies of MVCD spectra for high symmetry molecules that used both dispersive and FT-VCD instruments have been reported [113,114,115,116,117,118,119,120,121,122,123,124,125,126]. For moderate-size molecules in solution, such as substituted benzenes and porphyrins, and even metal carbonyls or C_60_, the spectra are dominated by couplet bandshapes, called A-terms, whose detection can often be enhanced by increasing resolution with FT-VCD [115,117,119,123], the B-term MVCD, due to field-induced mixing of different vibrations, is measurable, but usually is quite weak [5,126]. Since MVCD intensities scale linearly with magnetic field strengths, most experiments used instruments incorporating superconducting magnets with fields up to 8 T. However, one report did obtain measurable spectra of transition metal complexes using a fixed field permanent magnet, but in this case the intensity appeared to be enhanced by mixing with low-lying electronic states [5,127]. The effect of enhanced resolution, even up to ~0.1 cm^−1^, has a much greater impact on the MVCD of gas phase molecules, for which rotational lines can often be resolved and analyzed in terms of molecular magnetic moments [118,120,121,122,123,124,125]. For such rotationally resolved bands, the MVCD intensities can become large, particularly if the molecule is paramagnetic, even approaching ΔA/A~1 [127].

## 6. More Recent Developments

**FT-VCD.** While several companies now sell FT-VCD or VCD attachments to their FTIR spectrometers, most new method developments have come from Nafie’s lab and BioTools. Some of these are mentioned above, one of which relies on the corner-cube reflectors used in the Bomem interferometer that underlies that design. This instrument can use a dual source which naturally leads to more intensity at the detector and, in principle, improves S/N [5,29]. The two beams are 180° out of phase, meaning the signals tend to cancel for transmission measurements, so most FTIRs designed for measuring transmission could not adapt such a modification to their advantage. However, for VCD measurements, such a design reduces saturation of the detector and enhances the polarization modulated signal. This comes about because opposite polarizations are used for the two beams, which then makes them out of phase at the modulator. Combining these two independent phase relationships, interference and polarization, tends to sharply attenuate the centerburst, which reduces saturation, due to subtracting the intense transmission components of the signal, while summing their polarization modulation components (i.e., doubly out-of-phase is in-phase) leading to enhanced S/N. In this design, the two beams do not fully cancel, which provides a transmission interferogram that can be used for determining I_mod_/I_trans_ and hence ΔA, as has been evaluated in some detail by Nafie [5]. Proper scaling is obtained through calibration. Heating of the sample cell (which is open to the air) has been an issue reported by some users of this design. It is not clear if this is caused by the use of dual sources or by some other design aspect of this compact spectrometer. Presumably the sample cell temperature could be controlled by an external device, although this might be challenging in a small sample compartment. Such heating has not been an issue in external sample compartment designs.

Most FT-VCD experiments are done with Michelson interferometers, which are the standard in commercial FTIR instrumentation. Some exceptions have been reported. Polavarapu investigated the possibilities of using polarization division interferometry, wherein a grid polarizer is used for the beam splitter [128]. Depending on the grid used, this opens the possibility of going to much lower wavenumbers and can be used for circular dichroism measurements when an additional polarizer is in the beam. To date this has not developed further due to sensitivity limitations. A new method of polarization division interferometry with a common path has recently been developed by Polli and co-workers that uses a sliding wedge to create a variable delay between two polarizations that then interfere to give an elliptically polarized beam [129]. This approach has been shown to duplicate conventional VCD measurements as well as measure circular birefringence and to also operate with femtosec pulses opening the possibility of fast dynamic VCD measurements.

VCD spectra have long been plagued by artifacts, both as baselines with significant offsets from zero and as false signals that reflect the absorbance bands. Many modifications have been introduced to minimize these, including an initial polarization scrambling design of Cheng et al. [130], use of near collimated beams at the sample [72], use of lenses to remove reflection optics after the polarizer [39,51,52,53,72], and others. A more systematic approach has been developed by Nafie et al. [5,71,131], whereby a second PEM operating at a somewhat different modulation frequency is placed in the beam directly behind the sample. While this has some similarity to the earlier scrambling approach [130], it is much more developed, in that both modulated signals are measured and processed with lock-in amplifiers and the resulting interferograms are subtracted. If the design were to incorporate the same degree of modulation for both PEMs, the resulting difference interferogram would effectively remove the birefringent-induced signal from the measured VCD signal. In practice, the method greatly improves the baseline, as has been discussed in detail by Nafie [5]. An added component, insertion of a rotating half-wave plate in the beam after the second modulator, mimicking the methods developed by Hug to reduce artifacts in Raman optical activity (ROA) spectra [132], has been shown to have further beneficial baseline correction impact [133].

Lock-in amplifiers have long been the standard tool for demodulating signals with a fixed phase, time-varying component, such as induced by a PEM and a polarization sensitive sample. Such devices, often realized as analog boards containing just the needed features, have always been internal components of commercial CD spectrometers. Stand-alone lock-in amplifier instruments are widely available and have many fine qualities, but as we discussed above, the various designs and modifications for VCD instruments can require multiple lock-ins, and their use adds both expense and complexity with many features not really needed for the measurements at hand. More importantly, lock-ins often utilize analog filtering and can have problems of overload, or conditions where internal amplifiers become non-linear when used beyond ideal ranges. To simplify VCD spectrometers and better control signal quality, digital signal processing (DSP) was sought as an alternative to lock-in processing. One early approach used a step-scan interferometer with low frequency modulation of the mirror, then digitized and analyzed the entire signal at each step [70,77]. Data storage and higher frequency modulations were a challenge with this DSP approach, but by selecting various sidebands which could be reliably aliased to time scales appropriate for the digitizer, interferograms proportional to I_mod_ and I_trans_ were created in the computer. When ratioed and calibrated, I_mod_/I_trans_ gave normal ΔA results, and the spectra were of equivalent quality to conventionally obtained rapid-scan FT-VCD. They were better in quality than step-scan VCD obtained using a lock-in amplifier, which needed separate measurement of the transmission spectrum [70]. However, since the method used step-scan methods, data collection was slow compared to rapid scan FT-VCD, and there seemed to be little advantage to this style of DSP VCD. Alternatively, BioTools now takes a different approach and uses DSP processing of the full detector signal in their rapid-scan interferometer. This newer design can digitize all the needed signals without the use of any lock-ins by inserting new boards into their computer, which develops a VCD spectrum directly [5]. Unfortunately, details on how this data processing is implemented are not published (see: http://www.btools.com/chiralir2x.htm). Jasco now also promotes DSP processing in their VCD instrument.

**Alternate Laser-Based Methods.** Since detectors in the IR have nearly constant noise levels, it seems obvious that, to increase the S/N level, one should increase the source intensity. For the FT-VCD approach, this is somewhat limited by saturation issues, which the dual source modification addresses (see above). For dispersive instruments, one approach was to build a higher temperature black-body source, either a high temperature Nerst glower or a carbon rod, or for the near IR use of a tungsten-halogen lamp [18,20,21,24]. This dispersive approach has no saturation problems but, while useful, had only incremental impact on S/N.

Early on it seemed that the use of a laser source which could be tuned through the region of interest might provide a way to enhance S/N. Attempts to use diode lasers, which can tune over about 100 cm^−1^ by shifting gain between various modes that in turn are tuned by temperature and current variation, did not prove successful, in part because of mode hopping and the polarization properties of each line (Su, Keiderling, unpublished). In addition, the coherent properties of the laser beam meant that the modulator, due to surface reflections, acted as a Fabry–Perot interferometer whose internal path changes with the stress applied and consequently creates a large interference signal at the modulation frequency. Such a tunable laser approach to VCD has been addressed again by Lüdeke et al. [134,135] who used a quantum cascade laser (QCL) and took care to tilt the modulator and block the reflected beam with an aperture before the detector. Their results demonstrated that the QCL method can measure VCD and replicate FT-VCD results, but this did not initially show any obvious advantage. More recent results have used the QCL-VCD to do pH titrations of Pro, using higher frequency modulation (70 KHz) and Fourier filtering of the data [136]. Presumably such a QCL-based approach might more easily probe highly absorbing samples, such as biopolymers in water, or might eventually allow time-dependent experiments that follow VCD changes at fixed wavenumbers. 

Non-linear pump-probe experiments have also been shown to enhance S/N in IR experiments, particularly for very fast events. Two labs have explored the use of pulsed femtosecond (fs) lasers for VCD applications. Helbing and co-workers [137,138,139,140] used a dispersive design in which a momochromator selected the frequency bandpass of a probe beam whose VCD response was enhanced with heterodyne detection. The probe pulses were time-delayed to transit a PEM in phase with its maximum modulation (+ or −) and then passed through a sample and onto an MCT detector. The static (time-independent) VCD spectra matched published results well in the near IR for Ni and Co sparteine complexes. They also reported time-dependent pump-probe results that showed measurable changes at selected frequencies in the 10 s of a psec time scale.

An alternative approach was taken by Cho and coworkers [141,142,143,144,145], who linearly polarized a fsec beam and analyzed its polarization state with a second polarizer after passing through a chiral sample. While this sounds like an ORD measurement, they were able to use heterodyne detection to extract the IR and VCD in terms of a free induction decay (fid) and reproduce conventional experimental VCD results for β-pinene [145].

## 7. Spectral Analyses with Theoretical Modeling

This review is focused on instrumentation for polarization spectroscopy, which has been summarized above for the techniques of vibrational circular dichroism (VCD). Interpreting the data is a vital part of utilizing VCD in stereochemical applications, such as configurational and conformational analyses of chiral organic molecules, or biopolymer conformational studies. IR intensities are easy to compute in terms of the dipole strength (*D = **μ•μ***), where ***μ*** is the electric transition dipole moment. Conversely, due to fundamental difficulties in theoretical evaluation of the magnetic dipole component (***m***) of the rotational strengths (*R = **μ•m***) that correspond to the measured VCD intensities, it originally appeared that one would need to compute contributions arising from excited electronic states (i.e., go beyond the Born–Oppenheimer approximation) [4,5,6,9]. Consequently, initial efforts at simulating VCD spectra used empirical modeling approaches, such as dipole coupling approximations, that avoided such issues [144,145,146]. While occasionally useful, all these methods were recognized to be too simplistic to explain VCD spectra for all but a few ideal cases. Consequently, ab initio quantum mechanical (QM) models were sought to address the problem. One of these has become the standard for computing VCD in both academic and industrial labs [4,9,10,147,148]. As fully developed by Stephens, this method uses a magnetic field perturbation approach in the ground state to obtain the needed magnetic transition moment contributions of the motion of each atom, termed atomic axial tensor (AAT), that when coupled to similar atomic components of the electric transition dipole moments, that are used to compute IR spectra and termed atomic polar tensors (APT), were able to develop values for the VCD rotational strengths R. This method was extended to incorporate density functional theory (DFT), which improved both force fields (FF) or frequencies and intensities while increasing the speed and applicability of calculations [10,149]. It also added use of gauge-invariant orbitals to avoid origin dependence and has now been incorporated into several different QM packages (e.g., Gaussian, Cadpac, Dalton, ADF, and Turbomol), whose efficiency has increased markedly over the past decades [150,151].

For small- to medium-sized organic molecules (up to ~100 atoms), such DFT VCD calculations can be done directly on desk-top scale, multiprocessor, cluster-type computers (typically Linux-based). The simulated spectra constructed from DFT-calculated frequencies and AAT and APT intensities can be compared to measured VCD spectra, and conclusions can be drawn as to absolute configuration and favored conformations. This is a straightforward exercise for smaller molecules that have a rigid, single conformation. For these cases, the computed spectra normally agree in terms of overall sign patterns and rough intensities with what is measured, if the correct configuration was used, or the VCD will be mostly opposite in sign if the wrong configuration was used in the computation. Such comparisons are the basis of absolute configuration determination with VCD. However, for more flexible species, the main barrier becomes one of dealing with conformational equilibrium, since the VCD spectra can change dramatically as the conformation varies, while the IR changes are much smaller. After a complete conformational search, the structures of the lowest energy conformers can then be used to simulate VCD spectra. Usually several structures must be considered, since computed conformational energies normally have errors comparable to kT. One can then take different approaches depending on the conformational mix and spectral patterns that result. If there is a lowest energy predicted structure and its computed spectra agree with the observed spectra, while the others are quite different, then that selected configuration and conformation is favored. Usually several structures are in partial agreement with the data, and one must sort out configurational and conformational contributions, posing a more challenging process. If the configuration is known or favored for some independent reason, then averaging, or weighting by conformational energies, of spectra computed for low energy conformations sometimes can sometimes lead to improved agreement with experiment, and the result will suggest a conformational equilibrium. Thus, though computed VCD has become standard methodology, its applications for fluxional molecules still pose an interpretive challenge.

Biopolymer molecules offer a different challenge. Often such molecules are too large for reasonable computations at the DFT level, even with very large cluster machines. In addition, interactions with water, the normal solvent for biomolecules, are significant and can alter the spectra. One partially simplifying factor is that the absolute configurations of biopolymers are generally known and fixed, in that most peptides and proteins are composed of l-amino acids. Additionally, often the general or global conformation (helical, sheet-like, etc. on average) can be determined from CD or IR spectra. (Duplex nucleic acids are also often similarly restricted.) Full DFT simulations of VCD for moderately large peptides (up to ~25 residues) have been done, but often require restrictions on the side chains (conversion of residues to Ala is often used for simplification) (unpublished results from J. Cheeseman and T.A. Keiderling). To model spectra of even larger systems, Bouř and co-workers have developed a Cartesian tensor transfer method that computes FF and intensity parameters for smaller segments of the biopolymer and transfers them onto the larger structure [152]. Spectra for structures as large as globular proteins have been successfully simulated using this method [153]. Alternatively, isotope labeling can be introduced to the peptide sequence to spectrally shift the contributions of a local part of the biopolymer, and with computational modeling of VCD spectra for possible structural variants, local conformations can be deduced [100,154,155,156,157,158,159,160].

The problems of solvent interactions are more complex. It is possible to use polarized continuum models (PCMs) to partially correct the FF, but the effect on intensities is minor. Inclusion of discrete waters in VCD calculations has several problems, the first of which is knowing their structure, since the solvent is dynamic, the second of which is knowing how many waters to include, inner shell or more, and the last of which, significantly, is computing spectra for an effectively very large molecule once solvent molecules are included, which might be beyond the capabilities of resources available. Various reports that include discrete solvent effects have indicated some improvement in FF and thus frequencies and, for those peptides modeled, showed that the VCD intensities seem to shift along with the modes but do not change very much [161,162,163]. Thus, biopolymer VCD simulations often use vacuum or PCM-corrected calculations and adjust for the expected errors in frequencies under the assumption that the intensities will be qualitatively correct.

**Summary.** VCD instrumentation was first introduced a little over 40 years ago and, in the subsequent decade or two, was greatly enhanced in terms of sensitivity and spectra accessibility. The development of this polarization spectroscopy since then has primarily been in terms of applications, which have taken off, particularly in terms of relevance to the pharmaceutical industry and natural products analyses. Recent VCD instrumentation developments have been focused more on refinements and on making reliable commercial instrumentation widely available. Some new developments, which might offer the potential of measuring dynamic structural fluctuations with VCD, are on the horizon. There are many reviews and several books available and cited here that already provide numerous examples of VCD spectra and their applications. They thoroughly cover the field, so further repetition here seems superfluous; consequently, this review was restricted to a discussion of instrumentation and methods. VCD has become a developed and widely used spectroscopic method that has now passed into the accepted analytical repertoire with what is now a mature instrumentation and methodological basis.

## Abbreviations

AAT	atomic axial tensor
APT	atomic polar tensor
CD	circular dichroism
DC	direct current (non-oscillating signal)
DFT	density functional theory
FF	force field
FT	Fourier transform
IR	infrared
InSb	indium antimonide
MCT	mercury cadmium telluride
MVCD	magnetic vibrational circular dichroism
ORD	optical rotatory dispersion
PEM	photo elastic modulator
PMT	photo multiplier tube
QCL	quantum cascade laser
QM	quantum mechanical
S/N	signal to noise ratio
UV	ultraviolet
VCD	vibrational circular dichroism
